# Synthesis and Characterization of Quercetin–Iron Complex Nanoparticles for Overcoming Drug Resistance

**DOI:** 10.3390/pharmaceutics15041041

**Published:** 2023-03-23

**Authors:** Lucas Prestianni, Eric R. Espinal, Sarah F. Hathcock, Nadine Vollmuth, Pixiang Wang, Robert A. Holler, Shaoyang Liu, Brandon J. Kim, Yuping Bao

**Affiliations:** 1 Chemical and Biological Engineering, The University of Alabama, Tuscaloosa, AL 35487, USA; 2 Department of Biological Sciences, The University of Alabama, Tuscaloosa, AL 35487, USA; 3 Department of Chemistry and Physics, Center for Materials and Manufacturing Sciences, Troy University, Troy, AL 36082, USA; 4 Alabama Analytical Research Center, The University of Alabama, Tuscaloosa, AL 35487, USA; 5 Department of Microbiology, Heersink School of Medicine, The University of Alabama at Birmingham, Birmingham, AL 35487, USA; 6 Center for Convergent Biosciences and Medicine, The University of Alabama, Tuscaloosa, AL 35487, USA; 7 Alabama Life Research Institute, The University of Alabama, Tuscaloosa, AL 35487, USA

**Keywords:** iron complex, quercetin, drug resistance, flavonoids, efflux pump

## Abstract

Quercetin, one of the major natural flavonoids, has demonstrated great pharmacological potential as an antioxidant and in overcoming drug resistance. However, its low aqueous solubility and poor stability limit its potential applications. Previous studies suggest that the formation of quercetin-metal complexes could increase quercetin stability and biological activity. In this paper, we systematically investigated the formation of quercetin-iron complex nanoparticles by varying the ligand-to-metal ratios with the goal of increasing the aqueous solubility and stability of quercetin. It was found that quercetin-iron complex nanoparticles could be reproducibly synthesized with several ligand-to-iron ratios at room temperature. The UV-Vis spectra of the nanoparticles indicated that nanoparticle formation greatly increased the stability and solubility of quercetin. Compared to free quercetin, the quercetin-iron complex nanoparticles exhibited enhanced antioxidant activities and elongated effects. Our preliminary cellular evaluation suggests that these nanoparticles had minimal cytotoxicity and could effectively block the efflux pump of cells, indicating their potential for cancer treatment.

## 1. Introduction

Cancer remains a leading cause of death globally, and chemotherapy is still the most common treatment [1,2]. Unfortunately, tumor cells tend to develop resistance to multiple anticancer drugs, which greatly affects clinical outcomes [3]. Several mechanisms of drug resistance have been reported, such as drug efflux by overexpressed transporters (e.g., P-glycoprotein—P-gp), inhibition of the PI3K/AKT pathway, and activated cell growth factors [3]. Among these explored mechanisms of drug resistance, P-gp overexpression in cancer cells has been well accepted. This results in the exclusion of drugs from tumor cells, which prevents drug accumulation inside cancer cells [4,5,6]. Strategies have been developed to overcome drug resistance [7], such as inhibiting drug efflux transporters, structural alternation of drug molecules, gene delivery, etc. [8]. As natural antioxidants, flavonoids have attracted much attention for reversing multidrug resistance (MDR) to chemotherapeutics for cancer [7,9]. It is believed that flavonoids are involved in several regulatory processes, such as the regulation of drug efflux transporters, critical enzymes, oxidative stress, and cell cycles [7,10]. In particular, it has been shown that complexation with metal ions enhances the biological activities of flavonoids [11,12].

As one of the major naturally occurring flavonoids, quercetin (Q) has been extensively studied for cancer therapy [13,14,15,16]. For example, Q molecules have demonstrated activity in overcoming drug resistance, inducing cell apoptosis, and modulating tumor microenvironments in several cancer types [13,14,15,16]. Much evidence suggests that Q molecules are able to inhibit the efflux effects of several ATP-binding cassette (ABC) transporters, such as *ABCB1* (P-gp) [16,17], *ABCC1*, and *ABCC2* (MDR proteins 1 and 2) [11]. However, Q’s clinical applications in cancer therapy are greatly hindered by its extremely low aqueous solubility, poor stability from oxidation, and rapid enzymatic degradation, which lead to limited bioavailability [18,19]. To overcome these limitations, many efforts have been made to alter Q properties for enhanced biological activity [20], improved solubility [21], and improved bioavailability by forming protein complexes [22]. Additionally, the encapsulation of Q molecules inside nanocarriers has been explored for enhanced circulation time, increased bioavailability, and tumor targeting [23]. Particularly, complexation of Q with various metallic ions has exhibited enhanced biological activity, such as stronger antioxidant activity of Q metal complexes compared to free Q, [24,25], enhanced inhibitory effects on tumor cell growth, and increased bioavailability compared with free Q [26,27]. In addition, Q has multiple metal binding sites and can coordinate with various metallic ions to form complexes [24,28,29], such as cobalt, zinc, ruthenium, and iron. Compared to other complexes, iron (Fe) coordination has drawn special attention due to the demonstrated potential of Q-Fe complexes as MRI contrast agents [30]. Q-Fe complexes also exhibit the best antioxidant and antiacetylcholinesterase activities as promising agents for Alzheimer’s disease [24]. Therefore, the Q-Fe complex is explored in our study. Compared to Q-metal complex molecules, the formation of nanostructures based on Q-metal complexes led to additional benefits, such as nanoparticles for effective delivery [30], thin films and capsules for surface coating [31], and nanocomposites [32]. In our study, we aim at creating Q-Fe complex nanoparticles.

Here, we systematically investigated the feasibility of synthesizing Q-Fe complex nanoparticles (QFeNPs) under different conditions. In particular, the effects of Q-Fe ratios on NP formation, aqueous solubility, and stability were studied. Q molecules have distinct UV-Vis absorption bands, which were used to monitor the complex and QFeNP formation, study the stability, and quantify the solubility. The NP formation was also examined by transmission electron microscopy (TEM) for size and morphology, while the coordination between Q and Fe^3+^ was studied with Fourier-transform infrared spectroscopy (FTIR). Finally, we performed a preliminary evaluation of the inhibitory effects of these NPs on P-gp, which is expressed in human brain endothelial cells.

## 2. Materials and Methods

### 2.1. Chemicals

The majority of the chemicals were purchased from VWR, including quercetin dihydrate (97%, VWR, Aurora, OH, USA), iron (III) chloride anhydrous (FeCl_3_, 98%, VWR, Aurora, OH, USA), 2, 2-Bis (hydroxymethyl)-2, 2′, 2″-nitrilotriethanol (Bistris, ultra-pure, VWR, Ohio, USA), tris(hydroxymethyl)aminomethane (Tris, ultra-pure, VWR, Aurora, OH, USA), 10× phosphate-buffered saline (PBS) solution (VWR, Aurora, OH, USA), and ethanol (70%, VWR, Aurora, OH, USA). The following chemicals were purchased from other vendors as indicated, including sodium chloride (NaCl, 99%, Acros, Waltham, NJ, USA), methanol (99.8%, low acetone, Alfa Aesar, Haverhill, MA, USA), ultra-pure (4-(2-hydroxyethyl)-1-piperazineethanesulfonic acid) (HEPES) sodium salt (BDH, Radnor, PA, USA), and 1,1-Diphenyl-2-picrylhydrazyl free radical (DPPH, 97%, TCI, JP).

### 2.2. Synthesis of QFeNPs

A quantity of 5 mL of a 10 mM Q dihydrate methanol solution was mixed with 5 mL of a FeCl_3_ water solution of different molarities (50, 40, 30, 20, 10, 5, 3.3, 2.5, and 2 mM) to achieve Q-Fe ratios of 5:1, 4:1, 3:1, 1:1, 1:2, 1:3, 1:4, and 1:5. The reaction solutions were stirred at room temperature for 2 h. For temperature effects, experiments were performed with a Q-Fe ratio of 1:5, and the reaction solutions were stirred at room temperature at 37 °C and 60 °C. Then, the samples were processed for characterization and analysis. For TEM examination of NP formation and NP morphology, TEM samples were prepared by dropping 100 µL of an as-synthesized solution onto TEM grids. For UV-Vis spectrum collection, each as-synthesized solution (50 µL) was brought up to 5 mL with DI water and was then measured by a UV-Vis spectrometer. For solubility, stability, and activity tests, the reaction solutions were dialyzed in G2 Floatilizers for 4 h in sterilized DI water to remove methanol before freeze-drying. The dried powders were used for different analyses.

### 2.3. Stability of QFeNPs

The stability of QFeNPs was studied by monitoring their time-dependent UV-Vis spectra for up to two weeks at three different conditions: as-synthesized NPs in a 50/50 methanol/water solution, NPs stored in powder form after freeze-drying, and NPs in various buffers. For as-synthesized NPs, at day 1, day 7, and day 14, each 50 µL of the original NP solution was diluted into 5 mL of DI water and a UV-Vis spectrum was collected. For NP powders at the intervals of day 1, day 7, and day 14, 5 mg of QFeNPs for each ratio were dissolved into 1 mL of DI water and underwent 10 s of sonication. After further diluting to approximately 0.05 mg/mL with DI water, UV-Vis spectra were collected. For NPs in various buffers, ~5 mg of freeze-dried powders for each NP-forming ratio were dissolved into 1 mL of DI water, 150 mM of NaCl, 10 mM of Bis Tris, 10 mM of Tris, 10 mM of HEPES, and 1 × PBS under sonication. Then, 50 µL of the top solution at day 1, day 7, and day 14 were diluted into 5 mL of the respective buffer, and the UV-Vis spectra were collected.

### 2.4. Solubility of QFeNPs

The solubility of QFeNPs with clear and stable NP formations at Q-Fe ratios of 1:3, 1:4, and 1:5 was estimated. Specifically, standard curves of QFeNPs with Q-Fe ratios of 1:3, 1:4, and 1:5 were created using the UV-Vis absorbance at 293 nm, which was used to quantify the concentration of NPs at each ratio in different solutions. Approximately 5 mg of freeze-dried NP powders were dispersed into 1 mL of 1 × PBS, 10 mM of Bis Tris, 10 mM of Tris, 10 mM of HEPES, 150 mM of NaCl, or DI water, and each underwent sonication to create saturated NP solutions. Then, the UV-Vis spectra were collected on 50× diluted top supernatants after 24 h. The standard curves for each ratio were generated as follows: QFeNPs DI water solutions (~1 mg/mL) were created and then diluted to create concentrations within a range of 0.05 mg/mL and 0.005 m/mL, which were analyzed using a VWR V 3100PC UV-Vis spectrophotometer.

### 2.5. DPPH Antioxidant Activity

The antioxidant activity of the QFeNPs was measured using a DPPH antioxidant assay [33] and compared with free Q. Q and QFeNPs were dissolved in a water/methanol (50/50 *v/v*) mixture at various concentrations of 100, 75, 50, 25, and 10 μM. Then, 3 mL of a 0.1 mM DPPH solution was added to 1 mL of the sample solution while mixing. After 40 min, the absorbance of the solution was measured at 517 nm (A_s_). A quantity of 3 mL of DPPH (0.1 mM) was mixed with 1 mL of methanol and water and used as a control (A_0_). The absorbance of representative samples (100 μM) at 517 nm was also measured as a function of time, such as free Q and QFeNPs with a Q-Fe ratio of 1:5 and 2:1. The absorbance was recorded immediately after mixing and in 5 min increments for 30 min, after which absorbance was recorded at 1, 2, 4, and 6 h after mixing. The experiments were performed in triplicate, and the inhibition percentage of DPPH was then calculated using the following equation:

Inhibition (%) = 100 × (A_0_ − A_s_)/A_0_

### 2.6. Characterization

The morphology of QFeNPs was examined using a Hitachi H-7650 TEM (Hitachi, Ibaraki, Japan). The UV-Vis absorption spectra were collected in a range of 200–900 nm using 1 cm quartz cells on a VWR V 3100PC UV-Vis spectrophotometer. The FTIR spectra were recorded for powder samples in the range of 400–4000 cm^−1^ on an Agilent Cary 630 FTIR spectrometer (Agilent, Boulder, CO, USA). Freeze-drying was performed in a SP VirTis Benchtop Pro BTP-8ZL0VW freeze dryer (SP, Warminster, PA, USA). The heat-treated NP powder was analyzed by X-ray diffraction (XRD) on a Rigaku Miniflex with a Cu source at 30 kV and 15 mA. Thermogravimetric analyses (TGA) were performed on QFeNPs powders from freeze-drying using a TGA-550 (TA Instruments, New Castle, DE, USA) with a platinum-HT sample pan. Specifically, ~5 mg QFeNPs powder was placed into a platinum-HT sample pan, and the temperature was raised from room temperature to 800 °C at a heating rate of 10 °C/min. The measurements were carried out in a nitrogen environment with a gas flow of 60 mL/min for sample purge and 40 mL/min for balance purge.

### 2.7. P-gp Expression and Blocking Assay

Prior to studying the inhibition of QFeNPs on P-gp activity, we first evaluated the P-gp expression by immunostaining two types of cells: MDA-MB231br, a variant of triple-negative highly metastatic breast cancer cell lines, and human induced pluripotent stem-cell-derived brain-like endothelial cells (iBECs) [34,35,36,37]. Briefly, cells were fixed for 15 min with ice-cold methanol and blocked with 10% FBS in 1 × PBS for one hour at room temperature. P-gp antibody (Thermo Fisher Scientific, Waltham, MA, USA) was diluted 1:25 into a blocking solution, and cells were then stained overnight at 4 °C. After 24 h, the cells were washed three times with 1 × PBS, and a secondary antibody, Alexa-Fluor 488 mouse (Thermo Fisher Scientific, Waltham, MA, USA), diluted 1:200 in blocking solution, was added and incubated for one hour at room temperature, protected from the light. After incubation, the cells were washed twice with 1 × PBS, incubated with DAPI diluted at a ratio of 1:5000 in 1 × PBS for 15 min at room temperature in the dark, washed once more with 1× PBS, and then imaged on a TI2 Nikon microscope.

The effectiveness of QFeNPs on inhibiting P-gp activity was measured in iBECs using a fluorescent substrate Rhodamine 123 (R123) accumulation assay [38]. Cyclosporin A (CsA), a known P-gp inhibitor, was used as a positive control, while cells without treatment served as a negative control. The inhibition activity of three representative samples was tested, including free Q and QFeNPs with Q-Fe ratios of 2:1 and 1:5. All three samples were diluted to 100 µM and 50 µM in EC growth media without basic fibroblast growth factor (bFGF) or retinoic acid. Cells were incubated with the diluted samples for 2 h at 37 °C with 5% CO_2_. Then, cells were incubated with R123 (10 µM) with or without inhibitor and QFeNPs for 2 h at 37 °C with 5% CO_2_. Cells were then washed twice with PBS and lysed with 200 µL of RIPA buffer on a rocker for 10 min at room temperature, protected from light. The fluorescent intensity of R123 was measured on a Molecular Devices ID3 plate reader.

## 3. Results and Discussion

The objective of this study is to create QFeNPs with controlled sizes reproducibly and to increase Q’s aqueous solubility and stability in order to generate potential anti-drug resistance agents for cancer therapy. Prior to NP synthesis, we first investigated the pH-dependent structural stability of free Q using UV-Vis spectrometry and FTIR. Subsequently, the Fe^3+^ and Q complexation were studied by varying the Q-Fe ratios. For reactions with successful NP formation, we investigated the stability and aqueous solubility of QFeNPs. Finally, the inhibitory effects of QFeNPs were preliminarily tested on P-gp using iPSC-derived BECs in cell culture. Q was chosen as a coordination ligand for complex formation because of its proven anti-drug resistance activity and multiple binding sites [32]. The multiple binding sites allow adjusting the ratio of metal ions and further crosslinking for NP formation. The use of iron as the coordination metal ion is due to the potential of Fe complexes as MRI contrast agents [30]. Specifically, we studied the effects of temperature and Q-Fe ratios on NP formation with the goal of increasing Q’s aqueous solubility and stability.

### 3.1. Stability of Free Quercetin

**Figure 1:**
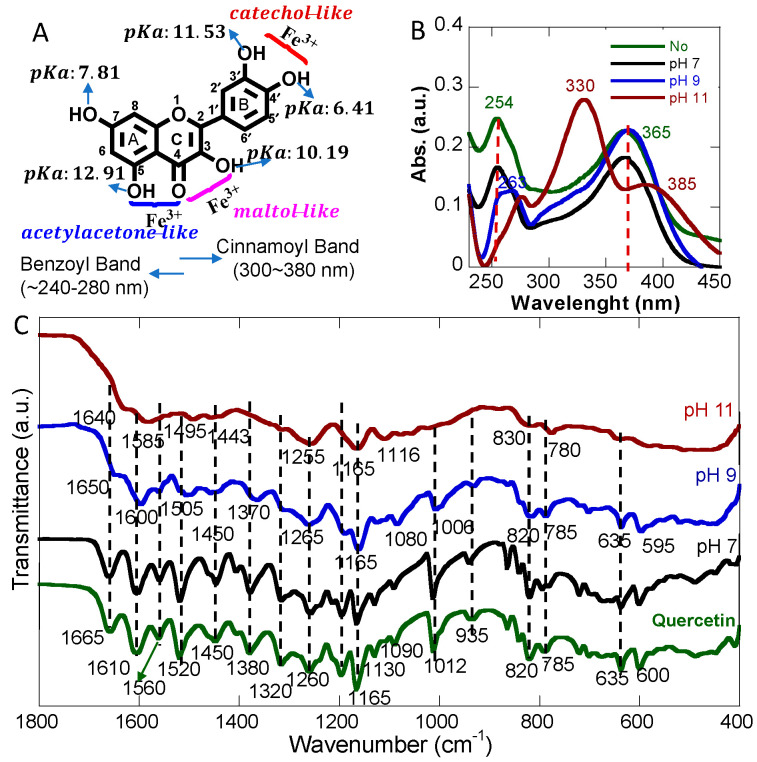
(**A**) Structure of quercetin with typical pKa values of hydroxyl groups and metal coordination sites along with typical absorbance, (**B**) UV-Vis absorption spectra of Q solution at different pHs compared with no pH adjustment, and (**C**) FTIR spectra of Q at different pHs compared with no pH adjustment.

As one of the major naturally occurring flavonoids, Q is known to have anti-drug resistance and anti-inflammatory activity, which is mainly attributed to its chemical structure [39]. Q has multiple metal binding sites [40], which allows adjusting Q-metal ion ratios for NP formation through crosslinking. Metal ions can coordinate through the catechol and carbonyl groups at the C4 position, assisted by hydroxyl groups at the C3 or C5 positions (Figure 1A), yielding catechol-like, maltol-like, and acetylacetone-like coordination interactions [40]. The functional groups have different pKa’s, leading to pH dependent stability and coordination behaviors. Although Q has five different pKas associated with its hydroxyl groups (-C4′-OH: 6.41, -C7-OH: 7.81, C3-OH: 10.19, C3′-OH: 11.53, and C5-OH: 12.91), Q methanol solutions at different pHs only exhibited two typical absorption peaks (Figure 1B). Without any pH adjustment, two typical absorbance bands were observed at 254 and 365 nm, which were consistent with reported studies with assigned B-ring cinnamoyl bands and A-ring benzoyl bands [30,41,42].

After adjusting the solution pH to 7 with 100 mM of NaOH, no changes in absorption were observed after 2 h at room temperature. Increasing the pH to 9, evident shifts were observed for both peaks (254 → 263 nm and 365 → 370 nm), likely due to deprotonation of some hydroxyl groups. In addition, a peak shoulder appeared around 300 nm, indicating some Q degradation. However, after pH was increased to 11, further shifts and decreases in intensities of the bands at 254 nm and 365 nm were observed. In addition, a major absorption band at 330 nm appeared, suggesting a significant structural change in Q. It has been previously reported that Q experiences rapid degradation at higher pH in solution, and the absorption band at 330 nm was mainly from degradation products, such as 2-(3,4-dihydroxyphenyl)-2-oxoacetic acid, as reported previously [43]. These degradation products showed three distinct bands at 274, 330, and 385 nm, with the 330 nm band being the most pronounced one [43].

The structural change of Q was also studied by comparing the alteration of functional groups based on the FTIR spectra of Q freeze-dried powders at different pHs. Similar to UV-Vis analysis, the FTIR spectra of free Q without pH adjustment and at pH 7 were identical. However, evident changes could be seen for pH 9 and pH 11 samples. The -OH stretching band around 3400 cm^−1^ showed significant broadening at higher pH, suggesting a structure change and the formation of likely carboxylic group-containing degradation products (Figure S1). Aromatic out-of-plane C-H bending vibrations at 820 and 785 cm^−1^ were not affected at pH 9 but broadened at pH 11, indicating ring-related changes. The bands at 600 and 635 cm^−1^ were likely associated with C-OH bending and ring stretching within ring C, which were not altered at pH 9 but completely disappeared at pH 11. Ring C breakdown is a direct indicator of Q degradation (Figure S2). In fact, previous studies have reported that the C3-OH position within ring C is considered the weakest point of Q and is susceptible to degradation [43]. The FTIR information of metal coordination site functional groups is mainly in the range of 1000–1800 cm^−1^, which is presented in Figure 1C. For Q powder prepared at pH 7, the FTIR spectra were identical and were consistent with previous studies with several characteristic peaks, including the band at 1665 cm^−1^ associated with the –C=O stretching at the C4 position and the C=C aromatic ring stretching bands at 1610, 1560, and 1520 cm^−1^ [44,45]. Increasing the pH caused Q degradation to start with the rearrangement and subsequent breakdown of ring C, leading to different single-ring degradation products (Figure S2). Briefly, the band at 1012 cm^−1^ could be attributed to C-CO-C stretching and bending in ketone at C4. This band did not change much at pH 9, suggesting the structural integrity of Q. However, this band completely disappeared at pH 11, indicating ring C breakdown and degradation of Q. Similarly, the 1200 cm^−1^ band refers to the C-O stretching in the aryl ether ring, which disappeared in the degradation products, consistent with the reported degradation products lacking the C-O ring [43]. The band at 1165 cm^−1^ could be assigned to the C-O stretching in phenol, which still existed in all degradation products, so the peaks were evident for all pHs tested. The stretching related to =C-O-H of the phenolic group was also observed at 1320 and 1380 cm^−1^ (OH in-plane bending). Both peaks broadened at pH 9, suggesting deprotonation. However, at pH 11, the peaks disappeared, indicating structural change. The aromatic ring stretching at 1260 cm^−1^ was not changed drastically at three different pHs because aromatic rings were also present in degraded products. Therefore, both UV-Vis and FTIR spectra suggest that Q molecules were relatively stable in methanol with no pH adjustment and at pH 7, but experienced degradation at pH 9 and pH 11. Therefore, all further studies were performed with Q in methanol without pH adjustment to minimize Q degradation.

### 3.2. Synthesis of QFeNPs

#### 3.2.1. Temperature Effects

**Figure 2:**
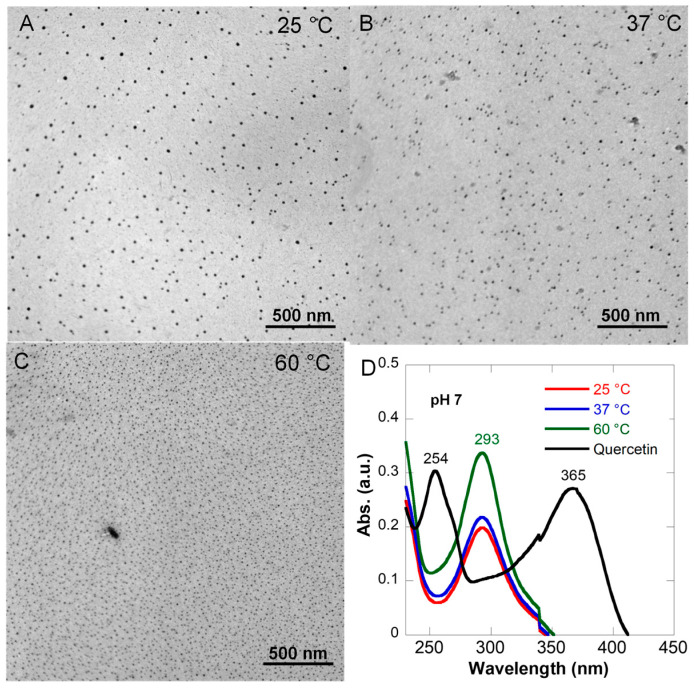
TEM images of QFeNPs prepared with a Q-Fe ratio of 1:5 at different temperatures: (**A**) 25 °C, (**B**) 37 °C, and (**C**) 60 °C, and (**D**) UV-Vis spectra of QFeNPs compared with free Q.

Previous studies suggest that higher temperatures promote Q degradation [46]. Prior to the study on the effects of different parameters on NP formation, we first tested the effects of reaction temperature on NP formation using a Q-Fe ratio of 1:5 and an excess amount of Fe for cross-linking. Three reaction temperatures were chosen: 25 °C, 37 °C, and 60 °C, to represent typical storage, application, and elevated temperatures. The Q-Fe interaction was mainly studied by UV-Vis spectroscopy. The morphology and size distribution were examined by TEM. NPs were formed at all three temperatures without much difference in morphologies, except for a slightly smaller size at a higher temperature (Figure 2). The UV-Vis spectra of QFeNPs prepared at different temperatures were almost identical, with a single peak around 293 nm, indicating similar coordination behaviors of Q and Fe. Comparing to previously reported molecular absorption of the Q-Fe complex with multiple peaks [26,47], QFeNPs only exhibited a single peak at 293 nm. The typical peaks of free Q at 254 and 365 nm were not observed, suggesting interactions of iron ions with both rings within the Q molecules. The higher absorbance of the sample made at 60 °C was likely due to the significant evaporation of methanol during the reaction. Even though NP formation was observed at all temperatures, all further reactions were performed at 25 °C to minimize Q degradation with increasing temperature.

#### 3.2.2. Q-Fe Ratio Effects

**Figure 3:**
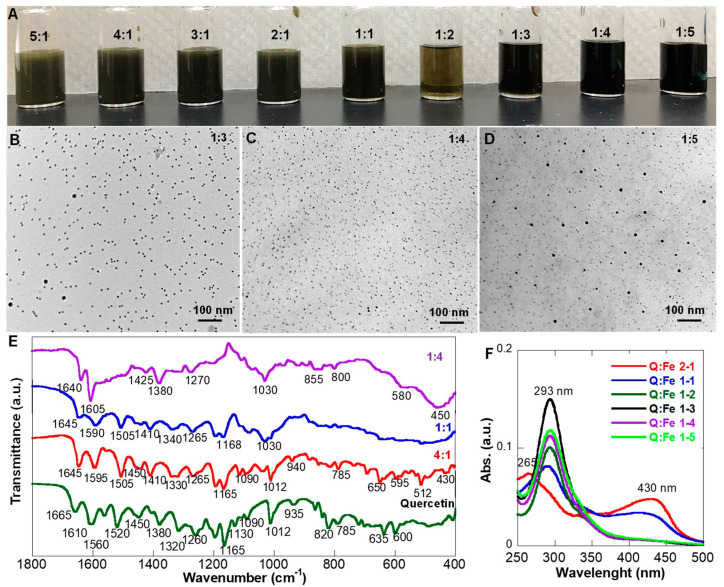
Ratio effect on NP formation: (**A**) photos of the reaction solutions at different ratios with different turbid levels; (**B**–**D**) representative TEM images of samples with NP formation at Q-Fe ratios of 1:3, 1:4, and 1:5; (**E**) FTIR spectra of representative samples with/or without NP formation; and (**F**) UV-Vis spectra of samples with NP formation at different ratios.

As illustrated in Figure 1A, a Q molecule has three iron coordination sites: catechol-like coordination on the B ring through catechol groups; maltol-like coordination through the hydroxyl group at C3 and the keto group on C4; and acetylacetone-like coordination through the C5-hydroxy- and C4-keto groups on the C ring. However, due to complex stability and steric hindrance when multiple molecules coordinate to a metal ion, the optimal ratio of Q to Fe for NP formation is difficult to predict. Therefore, the ratio effects on NP formation, aqueous solubility, and stability were investigated by varying the Q-Fe ratios from 5:1 to 1:5. The NP formation was monitored by examining the morphology of the products with TEM. The free Q methanol solution was light yellow, while the FeCl_3_ water solution was orange-brown. Upon mixing at room temperature, the reaction solution color changed to greenish-black, indicating a fast coordination process. After 2 h, the reactions were stopped, and the reaction solutions at different ratios exhibited variation in not only color but also translucency levels. Sample solutions with Q-Fe ratios of 5:1, 4:1, and 3:1 were completely turbid, while samples with Q-Fe ratios of 1:2–1:5 were translucent. However, samples with Q-Fe ratios of 2:1 and 1:1 were somewhere in between (Figure 3A). Further TEM analysis suggests a direct correlation between the formed products and their solution appearance. For example, with Q-Fe ratios of 5:1, 4:1, and 3:1, no NPs were formed; instead, large organic-like aggregate bundles were observed (Figure S4). With increasing Fe^3+^ amounts, NP formation was observed, and large aggregate bundles were barely seen at the 1:1 ratio. However, a dark TEM background was observed for samples with Q-Fe ratios of 1:2 and 1:1, suggesting that many of the Q molecules may exist as molecular Q-Fe complexes. Further decreasing the Q-Fe ratio, NPs were readily formed, as shown in Figure 3B,C. NPs with relatively uniform sizes around 6 nm were formed except for the 1:5 ratio, where a larger particle population around 10 nm was observed.

Based on the TEM observation, FTIR spectra were presented on selective samples representing the three types of products (bundles without NPs—4:1, some NP formation—1:1, and NPs—1:4) and compared with free Q (Figure 3E). The 4:1 ratio sample showed very similar IR bands to free Q, with a small band of Fe-O at 430 cm^−1^ and a visible decrease of the –C=O stretching band around 1560 cm^−1^, indicating some binding involvement of the –C=O group. The hydroxyl C-OH in-plane (1320 cm^−1^) and out-of-plane (785 cm^−1^) bending was clearly seen. In contrast, in the spectrum of the 1:4 ratio sample, the Fe-O band around 450 cm^−1^ was significant, and the –C=O stretching band around 1560 cm^−1^ was barely seen. Keto -C=O in-plane (1012 cm^−1^) and out-of-plane (820 cm^−1^) bands were shifted and broadened. Hydroxyl CO-H bending (1165 cm^−1^) became a broad shoulder. For the 1:1 ratio sample, the C=O in-plane (1012 cm^−1^) and out-of-plane (820 cm^−1^) bands were shifted and broadened, but the C=O stretching band around 1560 cm^−1^ was well reserved. The hydroxyl CO-H bending (1165 cm^−1^) was still clearly seen, suggesting Fe^3+^ ions were primarily interacting through the –C=O functional group. Hydroxyl C-OH in-plane (600 cm^−1^) and out-of-plane (785 cm^−1^) bending involved in the coordination was significantly altered at high iron concentrations. Previous studies on the Fe (II)–Q complex suggest Fe-O stretching occurs at ~630 cm^−1^ [26], which was absent in the FTIR spectra of free Q and QFeNPs. Therefore, the Fe^3+^ was not reduced. The spectral information suggests that coordination of metals occurred with the carbonyl group and OH linked to the C-3 carbon of Q.

Like the FTIR spectra, the UV-Vis spectra also depended on the Q-Fe ratios (Figure 3F). For samples with clear NP formation, such as Q-Fe ratios of 1:3, 1:4, and 1:5, only one major 293 nm absorption band was observed. In contrast, for 2:1 and 1:1 samples with a significant amount of molecular Q-Fe complex, two distinct peaks (at 265 and 430 nm) were observed related to a ligand-metal charge transfer, as reported by other studies [26,30]. For samples with evident NP formation, the absorption peaks of free quercetin at 254 and 365 nm disappeared. Instead, the UV-Vis spectra of QFeNPs exhibited a single peak around 293 nm, regardless of the reaction ratios of Q-Fe. However, the 2:1 ratio sample showed clear band shifts from free quercetin (254→265 nm and 365→430 nm) with a shoulder close to 290 nm. This observation is consistent with most previous studies in that the 2:1 Q:metal ratio provides the most favorable conditions for complex formation. The 420 nm peak also indicates the chemical reaction of iron (III) at the hydroxychromone site of Q, according to a previous report [47]. Our results are very different from a previous report using Q at pH 11 for NP formation as an MRI contrast agent [30]. Our pH-dependent stability assessment of free Q suggests that Q would be completely degraded at pH 11 and that the products would no longer be Q-Fe complex nanoparticles. Interestingly, after freeze-drying, QFeNP samples with all NP-forming ratios (2:1, 1:1, 1:2, 1:3, 1:4, and 1:5) only exhibited a single peak at 293 nm, indicating that the freeze-dried powder mainly contained QFeNPs (Figure S4A). The absorption peak around 430 nm from the as-synthesized samples with Q-Fe ratios of 2:1 and 1:1 disappeared. To understand whether the samples changed or the Q-Fe complex with absorption at 430 nm was removed during dialysis, several sample processes were conducted: samples before and after dialysis, samples with vacuum drying without dialysis, and water solutions of freeze-dried and vacuum-dried powders, as shown in Figure S4B,C. The presence of a Q-Fe complex absorbance for samples without dialysis suggests that the dialysis step removed the molecular complex.

### 3.3. Stability of NPs

We hypothesized that NP formation would increase the stability of free Q molecules because the Fe coordination at the weak site of the molecule is prone to degradation. Here, we investigated the stability of QFeNPs by monitoring the time dependent UV-Vis spectra of NPs at three conditions: as-synthesized NPs in a 50/50 methanol/water solution, NPs stored in powder form after freeze-drying, and NPs in various buffers. Special attention was given to the appearance of degradation products with absorptions around 330 nm, additional peak formation, and peak intensity changes at 293 nm. Our goal was to understand the effects of the relative Q-Fe ratios on NP stability. If the absorbance peak around 293 nm remained unchanged in terms of peak position and intensity, the NPs would be considered stable. Alternatively, if the absorbance of the NP solution changed greatly with additional absorption peaks, it would be attributed to Q degradation.

**Figure 4:**
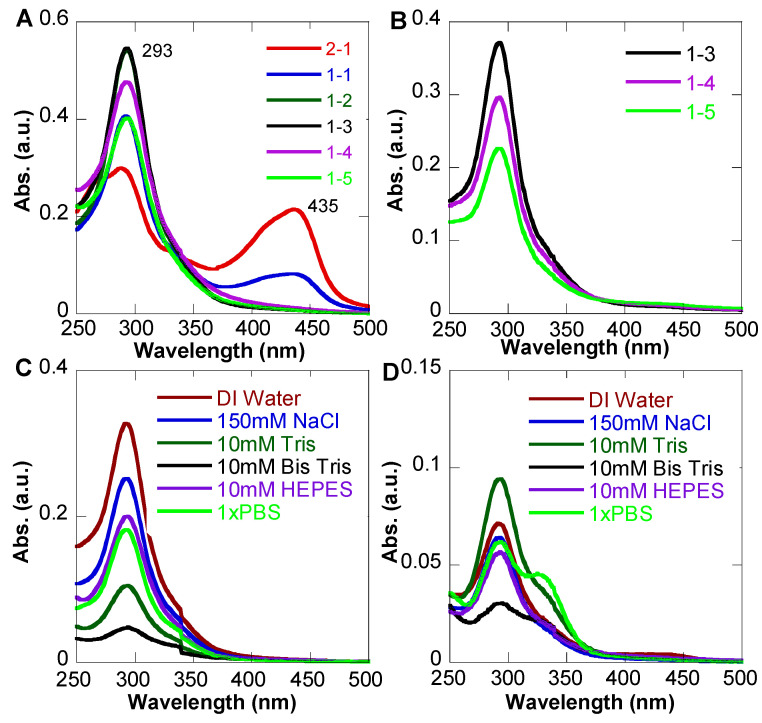
UV-Vis spectra of QFeNPs at various conditions after two weeks: (**A**) as-synthesized NPs at various ratios in 50/50 methanol/water; (**B**) NP powders with Q-Fe ratios of 1:3, 1:4, and 1:5 after freeze-drying; (**C**) QFeNPs with a Q-Fe ratio of 1:4 in different solutions; and (**D**) QFeNPs with a Q-Fe ratio of 2:1 in different solutions.

Figure 4A shows the UV-Vis spectra of as-synthesized samples in 50/50 methanol/water solutions after two weeks. We observed that NP formation samples only showed relative intensity changes compared to Figure 3F. The absorption at 293 nm of 2:1 and 1:1 ratio samples became evident, and the 254 nm peak completely disappeared. In addition, the 430 nm peak is slightly redshifted with increased intensity. No peaks indicating degradation of the free Q peak were observed for all samples. Therefore, the NP formation did increase the stability of free Q. Once QFeNPs were formed and freeze-dried, they were very stable in powder form. Figure 4B shows the UV-Vis spectra of freeze-dried QFeNPs redispersed in DI water, where the peak position was not changed after two weeks. However, once the QFeNPs were dispersed in different buffers, a variation in stability was observed depending on the Q-Fe ratios. Figure 4C shows the representative UV-Vis spectra of QFeNPs with a Q-Fe ratio of 1:4, where the NPs were stable in all six tested conditions but had the lowest solubility in the Bis-Tris buffer. In contrast, the samples with a Q-Fe ratio of 2:1 showed clear degradation after two weeks in PBS, Bis Tris, and HEPES, with a degradation peak around 330 nm (Figure 4D). This observation could be attributed to a competing interaction with Fe^3+^ in the Q-Fe complex.

### 3.4. Solubility of NPs

**Figure 5:**
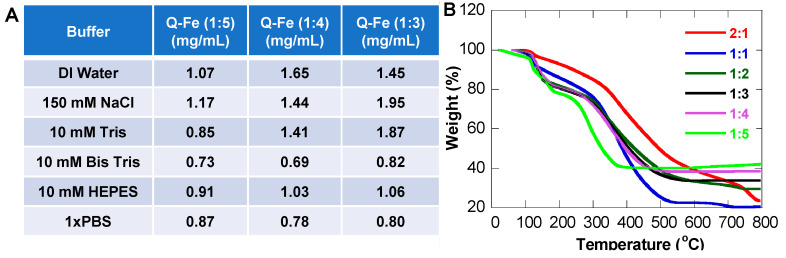
(**A**) Solubility of QFeNPs with Q-Fe ratios of 1:5, 1:4, and 1:3 in various solutions; and (**B**) TGA plots of QFeNPs powders with all Q-Fe ratios after freeze-drying.

Another goal of NP formation was to increase Q solubility in aqueous solutions. Most commercial vendors classify free Q as insoluble in aqueous solution. A few studies suggest that free Q in water has a solubility of 2–4 µg/mL, which does not reach the therapeutic threshold suggested by in vitro and in vivo studies in the order of tens micromolar [48,49]. Metal complex formation has clearly demonstrated increased biological activity, but no clear solubility information can be found. Our stability studies of QFeNPs also showed different absorption intensities, suggesting a solubility variation in different solutions. Here, we quantified the solubility of QFeNPs with clear and stable NP formation at Q-Fe ratios of 1:3, 1:4, and 1:5 in various solutions. Specifically, standard curves of QFeNPs at Q-Fe ratios of 1:3, 1:4, and 1:5 were created using the UV-Vis absorbance at 293 nm, which was used to quantify the concentration of NPs at each ratio. The table in Figure 5A presents the average solubility of QFeNPs from three tests by creating a saturated solution (5 mg/mL) and measuring the top absorbance after 24 h. DI water and 150 mM salt conditions exhibited a higher solubility compared to Bis-Tris and PBS. Tris and HEPES fell in between. The solubility of QFeNPs at all ratios was increased compared to free Q.

In order to understand the Q-Fe ratio-dependent behaviors, TGA experiments were performed on freeze-dried QFeNPs in a nitrogen atmosphere in the temperature range from room temperature to 800 °C at a heating rate of 10 °C/min. As expected, NPs with different Q-Fe ratios exhibited distinct weight loss onsets and different decomposition behaviors, as shown in Figure 5B. No weight loss was observed for all samples below 100 °C, suggesting a lack of surface-adsorbed water molecules. The weight losses below 200 °C were mainly from complex or Fe-bound water molecules [26,50]. Depending on the Q-Fe ratios, the bound water molecules varied, and the onsets of Q decomposition were also different, with the 1:5 ratio sample being the lowest at around 250 °C until a stable weight was reached. When the weight reached a plateau, Q decomposition was complete, and iron formed inorganic compounds. X-ray diffraction analysis of QFeNP powders after heating up to 450 °C for 4 h suggests a hematite iron oxide (Fe_2_O_3_) phase (Figure S5), where the Fe content (70%) and O weight percentage (30%) were used to estimate the Q-Fe ratios within QFeNPs. The detailed information is presented in Table 1. For samples with a Q-Fe ratio of 2:1, the H2O-Q-Fe ratio could not be obtained because the sample continued to decompose beyond 800 °C, a similar degradation behavior to free Q molecules [24]. With increasing Fe content to 1:1 and 1:2 ratios, the sample decomposition showed two weight loss windows: a window in the range of 300–500 °C likely associated with the Q-Fe complex and another higher temperature window starting at 700 °C from the complete decomposition of free Q. For higher Fe contents, the Q decomposition only had one window with a lower stated temperature at higher Fe contents, suggesting that the Fe coordination decreased the decomposition temperature of the complex with Q-Fe ratios of 1:3, 1:4, and 1:5 which correspond to inorganic compound formation around 575, 500, and 400 °C, respectively. The calculated ratio of H2O-Q-Fe for all the samples suggests that the bound water molecules kept increasing when the Q-Fe ratios were greater than 1:1, but the Fe coordination only became saturated for the 1:5 Q-Fe ratio. Our TGA analysis (Table 1) suggests an increase in water coordination with NP formation, which could be correlated with the stability and solubility of QFeNPs.

### 3.5. Antioxidant Activity of QFeNPs

**Figure 6:**
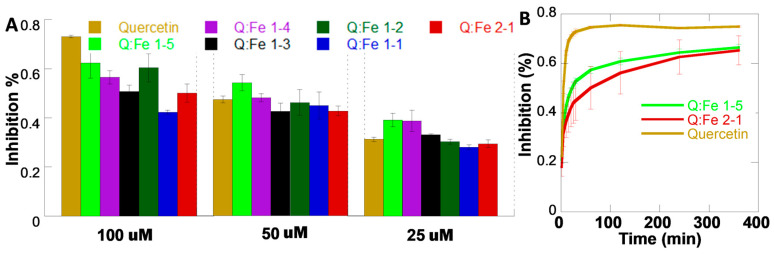
DPPH assay: (**A**) inhibition (%) of DPPH by free Q and QFeNPs with various Q-Fe ratios after 40 min at 100 μM, 50 μM, and 25 μM; and (**B**) time-dependent inhibition (%) of DPPH by free Q and QFeNPs with Q-Fe ratios of 2:1 and 1:5 at 100 μM.

Free Q and Q-metal complexes are known to have antioxidant activities [24,25]. To study whether NP formation affects the antioxidant activity, the DPPH assay [33] was performed on QFeNPs with various Q-Fe ratios at 25, 50, and 100 μM, and the inhibition (%) was calculated and compared with free Q (Figure 6). DPPH exists in a stable free radical form at room temperature and has a strong absorbance at 517 nm. In the presence of an antioxidant molecule, DPPH will be reduced to a colorless form. Thus, the DPPH assay provides an easy way to assess the antioxidant activity of compounds by monitoring DPPH inhibition [33]. Here, we observed DPPH inhibition for QFeNPs with all ratios at three concentrations (Figure 6A). In general, the percentage of DPPH inhibition increased with increasing NP concentrations. However, compared to free Q controls, QFeNPs at lower concentrations (25 and 50 μM) showed greater antioxidant activity. However, at 100 μM, free Q exhibited higher antioxidant activity. Previous studies on Q-metal complexes with 2:1 Q-metal ratios suggest that the antioxidant activity of the complex might be metal ion dependent, similar to the lower antioxidant activities for Sb (II) and Tb (III) Q complexes [50,51] and the higher antioxidant activities for Co (II) and Pb (II) Q complexes [52,53,54]. The key difference of our samples from reported studies is the NP formation. We hypothesize that the NP formation could potentially affect the release rate of free Q molecules and consequently elongate the time of action. Therefore, time-dependent DPPH inhibition was performed on representative samples at 100 μM, such as QFeNPs with Q-Fe ratios of 2:1 and 1:5, compared with free Q controls for up to 6 h (Figure 6B). Within the first 30 min, the inhibition was measured every 5 min, then the inhibition was measured at 1, 2, 4, and 6 h. After around 25 min, free Q reached maximum DPPH inhibition, while the QFeNPs showed continuous inhibition and did not reach maximum inhibition after 6 h. This observation confirmed our hypothesis that NP formation can increase the release time of free Q.

### 3.6. Inhibitory Activity of QFeNPs on P-Glycoprotein Function

**Figure 7:**
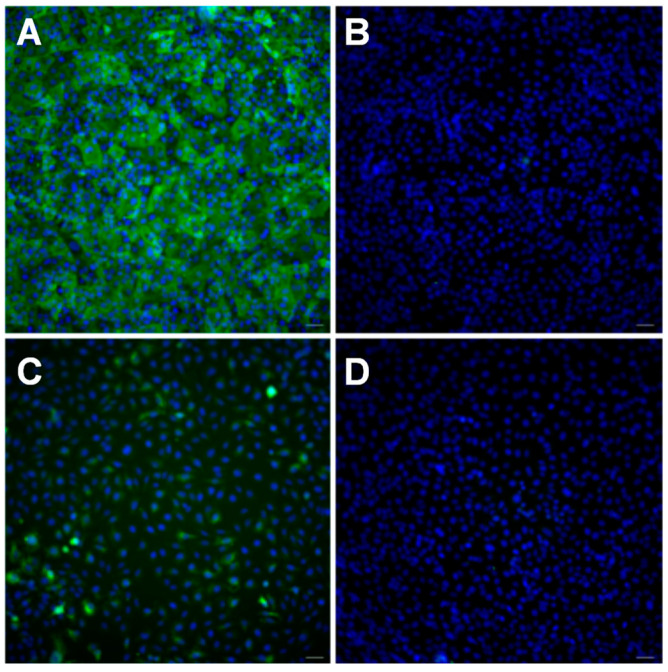
The immunostaining of P-gp on (**A**) iBECs labeled with P-gp antibody Alexa-488 tagged secondary antibody; (**B**) iBECs labeled only with Alexa-488 tagged secondary antibody; (**C**) MDA-Mb-231 Br labeled with P-gp antibody Alexa-488 tagged secondary antibody; and (**D**) MDA-Mb-231 Br labeled only with Alexa-488 tagged secondary antibody. Cells were observed using a 20× objective. Cell nuclei were stained with DAPI blue.

P-gp is an ABC membrane MDR transporter that acts as a drug efflux pump and is encoded by the gene *ABCB1* [6]. This membrane protein is known to be overexpressed in various cancer cells [6]. For example, the triple-negative and highly metastatic breast cancer cell line MDA-MB-231, after developing drug resistance, showed a much higher level of P-gp expression [55,56]. In the human body, various tissues and organs related to drug pharmacokinetics also physiologically express P-gp, such as the intestine, liver, kidney, and brain endothelial cells of the blood–brain barrier [57]. Therefore, P-gp is directly involved in the regulation of drug intestinal absorption, brain tissue penetration, and liver and renal accumulation and serves as an important factor of the drug pharmacokinetics [57]. Previous studies have shown that BECs not only have functional P-gp expression but are also able to pump out a wide range of molecules [58,59]. The functional expression of MDR transporters in BECs was also reported [60]. First, we evaluated P-gp expression through immunostaining on two types of cells: MDA-MB231br, a variant of MDA-MB 231, and iPSC-derived BECs (iBECs), as shown in Figure 7. Cells were labeled with the anti-P-gp antibody, followed by the Alexa-488-labeled secondary antibody. Cells without the P-gp antibody treatment were used as a comparison. Significant green fluorescence was observed for P-gp antibody-labeled iBECs (Figure 7A) compared to control cells (Figure 7B), suggesting a high level of expression of P-gp in iBECs. In contrast, green fluorescence was also observed for the P-gp antibody-labeled MDA-MB-231br cells (Figure 7C) compared to the control cells (Figure 7D). However, the fluorescence levels were significantly lower than those of iBECs. This is consistent with the literature reports that only the selected resistant breast cancer cells exhibit significantly higher P-gp expression, in contrast to the original cell lines [56,57]. We have also performed viability assays on these two types of cells: MDA-Mb-23br and iBECs, to study the cytotoxicity of QFeNPs (Figure S6). Here, cells were treated with representative QFeNPs at Q-Fe ratios of 2:1 and 1:5 at 50 and/or 100 µM. Cell viability was presented as the percentage of live cells using untreated cells as a control. We did not observe a significant alteration in cell viability for both cell types. Cell viability was slightly altered in some cases, such as cells treated with 50 and 100 µM QFeNPs with Q-Fe ratios of 2:1 (Figure S6A), but statistical analyses showed that the variation was not drastic in iBECs. The viability of iBECs was similar for all treatments (Figure S6B).

**Figure 8:**
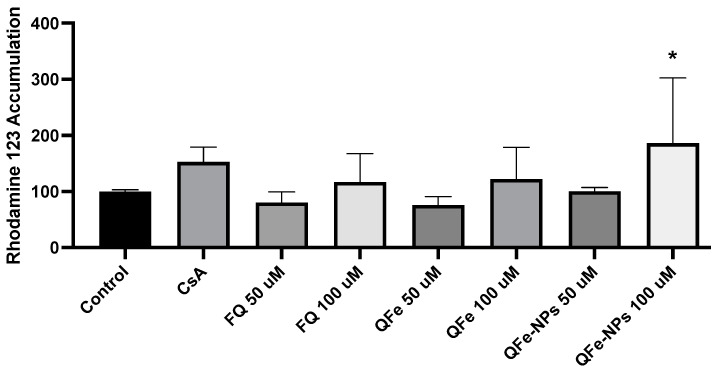
Rhodamine 123 accumulation inside BECs treated with free quercetin (FQ), the QFe complex with a 2:1 Q-Fe ratio, or QFeNPs with a 1:5 Q-Fe ratio at 50 µM and 100 µM along with CsA, as a positive control, and cells without treatment as a negative control. Data are presented as mean values from two independent iPSC-derived BEC differentiations conducted in triplicate (*n* = 6). Error bars represent SD. An ANOVA was used to determine significance compared to the control. * *p* < 0.05.

Because of the higher expression levels of P-gp in iBECs, as shown, iBECs were chosen as a model system to investigate the use of QFeNPs to modulate the function of P-gp in BECs. We tested three representative samples: free Q, samples with a Q-Fe ratio of 2:1, which formed stable complex molecules, and samples with a Q-Fe ratio of 1:5, which formed QFeNPs. The P-gp function was measured using a fluorescent substrate R123 accumulation assay [33]. The accumulation of the substrate inside the cells is an indication of the blockage of the P-gp efflux pump. We observed a trend toward increased R123 accumulation with higher concentrations of all QFeNPs (Figure 8). The R123 accumulation inside cells treated with 100 µM QFeNPs with a Q-Fe ratio of 1:5 showed statistically significant but higher R123 accumulation. Even though further detailed investigation is needed, our preliminary results suggest the contribution and effectiveness of QFeNPs in regulating P-gp function in BECs.

## 4. Conclusions

In conclusion, QFeNPs have been successfully synthesized with several Q-Fe ratios. The Q-Fe ratios directly affected NP formation, but UV-Vis absorbance and TGA analysis suggest that only a 1:5 Q-Fe ratio achieved full coordination of all three binding sites of Q. The QFeNPs at all Q-Fe ratios exhibited increased stability and aqueous solubility when compared to free Q. For example, QFeNPs with Q-Fe ratios of 1:3, 1:4, and 1:5 showed increased solubility (>1 mg/mL) in DI water and 150 mL of NaCl. Stability monitoring using UV-Vis spectroscopy suggests that close to full coordination of Q-Fe (1:3) made NPs more stable in buffers. For example, QFeNPs showed clear degradation in PBS and tris buffer, likely because buffer molecules (e.g., phosphate and amine groups) could interact with Fe, leading to Fe dissociation from the QFeNPs. The cellular viability tests indicated that these NPs had minimal cytotoxicity. Our preliminary cellular testing suggests that the Q-Fe nanoparticles could effectively inhibit the P-gp efflux pump in cells with high levels of P-gp expression. Therefore, these NPs have great potential for overcoming several pharmacokinetic limitations of free Q and opening possibilities for multiple purposes and different applications. The proposed NPs are based on dietary natural products for overcoming MDR in cancer treatment and have great potential for cancer prevention with less safety concern and better public acceptance. This system can be used in combination with multiple drugs to reduce side effects and increase overall efficacy and safety. Our future studies will focus on QFeNP’s effectiveness in overcoming drug resistance in different drug-resistant cell lines and further understanding the anti-cancer mechanisms of natural products that can help prevent and manage many types of cancer.

## Figures and Tables

**Table 1:** TGA analysis of nanoparticles with different Q-Fe ratios.

Sample	2:1	1:1		1:2		1:3		1:4		1:5	
Dehydration (°C/%)	112–131	110–131		118–149		120–155		116–160		113–124	159–181
	4%	13%		20%		20%		20%		10%	10%
Q Decomposition (°C/%)	-	312–463	653–715	299–491	702–747	302–471		290–442		258–345	
		64%	2%	49%	3%	46%		54%		38%	
Inorganics (°C/%)	N/A	>750 °C	6% O	>750 °C	8% O	>575 °C	10% O	>500 °C	11% O	>400 °C	13% O
			15% Fe		20% Fe		24% Fe		26% Fe		28% Fe
H_2_O:Q:Fe	-	3:1:1.1		5.5:1:1.8		6:1:2.3		6.2:1:2.6		6.6:1:3	

## Data Availability

Not applicable.

## Supplementary Materials

The following supporting information can be downloaded at: https://www.mdpi.com/article/10.3390/pharmaceutics15041041/s1, Figure S1: FTIR spectra of free quercetin at different PHs in the range of 2000–4000 cm^−1^; Figure S2: Quercetin degradation process and major degradation products; Figure S3: TEM images of products formed at different Q-Fe ratios; Figure S4: UV-Vis spectra of samples with Q-Fe ratios of 2:1 and 1:2 at different processing conditions; Figure S5: XRD spectra of QFeNPs powder heated at 450 °C under argon for 2 h; Figure S6: Cell viability.
