# Supplementary material for: Synthesis and Characterization of Quercetin–Iron Complex Nanoparticles for Overcoming Drug Resistance
Source: Pharmaceutics. 2023 Mar 23;15(4):1041. doi: 10.3390/pharmaceutics15041041 (PMC10144594; doi:10.3390/pharmaceutics15041041)
Supplement: Supplementary file 1 [file pharmaceutics-15-01041-s001.zip › pharmaceutics-2199969-supplementary.pdf]

Synthesis and Characterization of Quercetin–Iron Complex Nanoparticles for Overcoming Drug Resistance

Lucas Prestianni [1], Eric Espinal [2], Sarah F. Hathcock,[2] Nadine Vollmuth,[2] Pixiang Wang [3], Robert A Holler [4], Shaoyang Liu [3], Brandon J. Kim [2,5,6,7*] and Yuping Bao [1,6,7,*]

[1] Chemical and Biological Engineering, The University of Alabama, Tuscaloosa, AL, 35487, USA.
[2] Department of Biological Sciences, The University of Alabama, Tuscaloosa, AL, 35487, USA.
[3] Center for Materials and Manufacturing Sciences, Department of Chemistry and Physics, Troy University, Troy, AL, 36082, USA.
[4] Alabama Analytical Research Center, The University of Alabama, Tuscaloosa, AL, 35487, USA.
[5] Department of Microbiology, Heersink School of Medicine, The University of Alabama at Birmingham, Birmingham, AL, 35487, USA.
[6] Center for Convergent Biosciences and Medicine, The University of Alabama, Tuscaloosa, AL, 35487, USA.
[7] Alabama Life Research Institute, The University of Alabama, Tuscaloosa, AL, 35487, USA.
* Correspondence: ybao@eng.ua.edu; bjkim4@ua.edu

1. *FTIR of quercetin at different pH*

Figure S1 showed the FTIR spectra of free quercetin at different pHs in the range of 2000-4000 cm^{-1}, where the -OH stretching band around 3400 cm^{-1} showed increased intensities and significant broadening, consistent with the degradation of quercetin at pH 11. The formation of different carboxyl groups would lead to a stronger absorbance in this region.

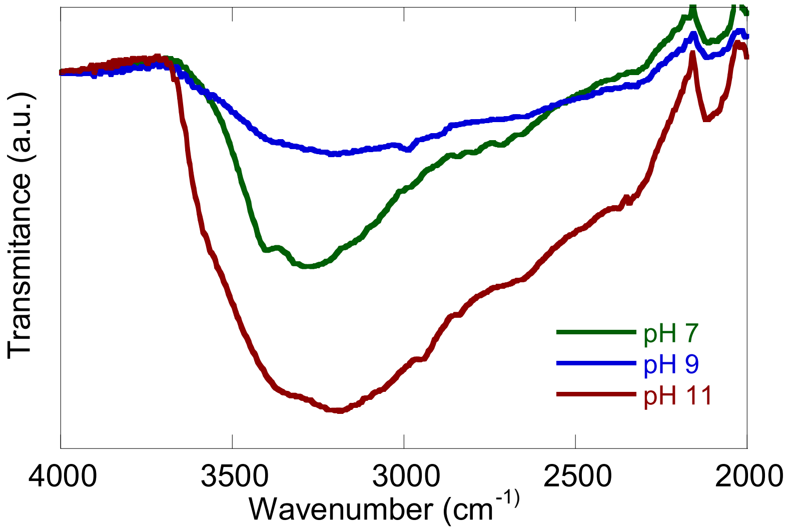

Figure S1. FTIR spectra of free quercetin at different pHs in the range of 2000-4000 cm^{-1}.

2. *Degradation of Quercetin*

Ring C break-down is believed to be the typical degradation process of quercetin with the C3-OH position within ring C being the weakest point prone to degradation [43]. The major degradation products consist of carboxylic groups, which increase its solubility and stability.

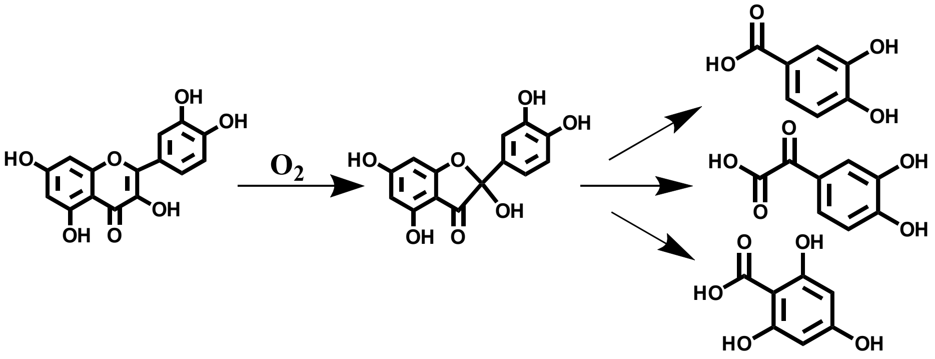

Figure S2. Quercetin degradation process and major degradation products.

3. *Nanoparticle formation at different ratios*

Because of the defined available binding sites within a single molecule, not every Q-Fe ratio led to NP formation. At higher Q-Fe ratios (5:1, 4:1, 3:1), the quercetin molecules aggregated into bundles with no NP formation. With increasing the Fe concentration, NP formation was observed, as shown in Figure S3.

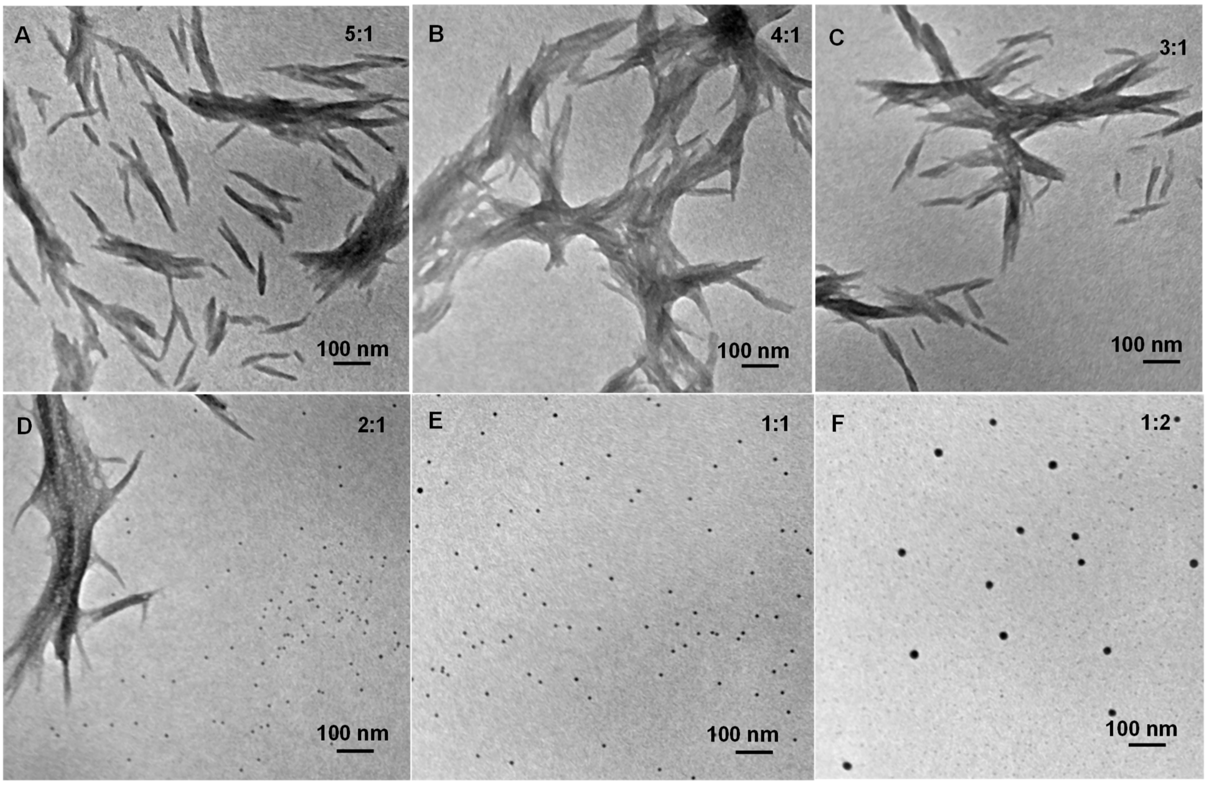

Figure S3. TEM images of products formed at different Q-Fe ratios.

4. *Stability of nanoparticles*

After freeze-drying, the UV-vis spectra of samples with Q-Fe ratios of 2:1 and 1:1 only exhibited the NP absorption peak at 293 nm, and the absorption peak around 430 nm from the as-synthesized samples disappeared. To understand whether the samples changed or the Q-metal complex with absorption at 430 nm were removed during dialysis, several sample processes were conducted: samples before and after

dialysis, samples with vacuum drying without dialysis, and water solution of freeze-dried and vacuum-dried powders, as shown in Figure S4. The presence of Q-metal complex absorbance for samples without dialysis suggested that the dialysis step removed the complex.

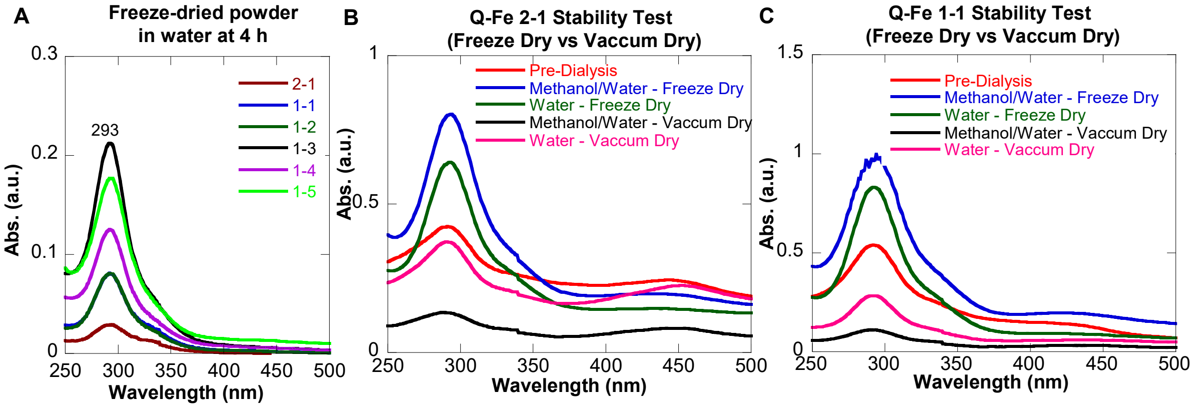

Figure S4. UV-vis spectra of samples with Q-Fe ratios of 2:1 and 1:2 at different processed conditions.

5. *Solubility of nanoparticles*

To best estimate the solubility, several standard curves were created and compared: absorbance peak area, intensity difference between peak absorbance and left peak starting absorbance, and intensity difference between peak absorbance and right peak ending absorbance. The peak curve gave the best linear fit and went through origin; therefore, the peak area was used to create the standard curve for estimating the solubility of the QFeNPs in different solutions.

6. *XRD of the QFeNPs treated at high temperature*

In order to estimate the iron content in each sample, the inorganic powder, after heating at an inert gas atmosphere for 2 h, was analyzed by x-ray diffraction (XRD) on a Rigaku Miniflex with a Cu source at 30 kV, 15 mA. Figure S5 shows the XRD pattern of the powder, which matched well with the hematite iron oxide (Fe2O3) phase, where the Fe was used to calculate the Fe content in the QFeNPs. The O weight percentage was added to the weight loss of the organic competent during TGA analysis.

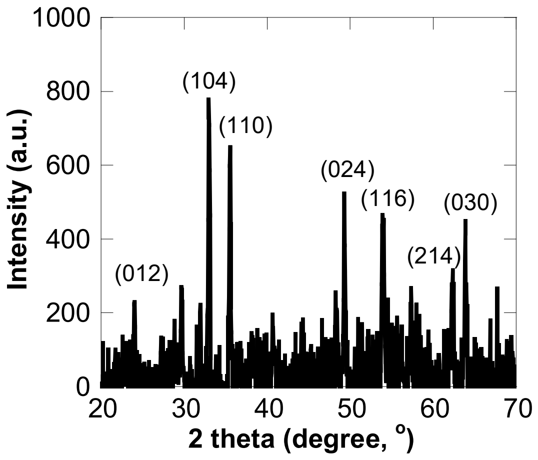

Figure S5. XRD pattern of QFeNPs powder heated at 450 °C under argon for 2 hours

7. Cytotoxicity of QFeNPs

To study the cytotoxicity of the QFeNPs, we performed viability assay on two types of cells: MDA-Mb-23br, a triple negative high metastatic breast cancer cell line, and human induced pluripotent stem-cell (iPSC) derived brain-like endothelial cells (BECs), as shown in Figure S6. In brief, cells were incubated with QFeNPs of Q-Fe ratios of 2:1 and 1:5 at 50 and/or 100μM to test cytotoxic effects. After 5h incubation, cells were detached with 1x Trypsin (Corning), mixed with Trypan blue (Gibco) 1:1 and transferred onto a countess counting slide. Live and dead cells were counted by the automated cell counter Countess 3 (Thermo Fisher Scientific). Cell viability was presented as percentage of live cells, and statistical analysis was done via GraphPad one-way ANOVA. Cells treated with a water vehicle were used as controls. Compared to control cells, we did not observe significant alteration in cell viability for both cell types. Cell viability was slightly altered in some cases, such as cells treated with 50 and 100 μM QFeNPs with Q-Fe ratios of 2:1 (Figure S6 A), but the statistical analysis showed that the variation was not drastic in BECs. The viability of BECs was similar for all treatments.

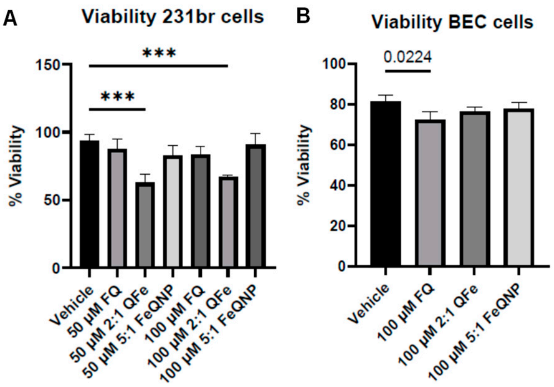

Figure S6. Viability test of MDA-MB231br cells and BEC cells treated with free Q, and QFeNPs with Q-Fe ratios of 2:1 and 1:5 at 50 and 100 μM.
